# Investigating the Reliability of Population Receptive Field Size Estimates Using fMRI

**DOI:** 10.3389/fnins.2020.00825

**Published:** 2020-07-30

**Authors:** Agustin Lage-Castellanos, Giancarlo Valente, Mario Senden, Federico De Martino

**Affiliations:** ^1^Department of Cognitive Neuroscience, Faculty of Psychology and Neuroscience, Maastricht University, Maastricht, Netherlands; ^2^Department of NeuroInformatics, Cuban Center for Neuroscience, Havana, Cuba; ^3^Center for Magnetic Resonance Research, Department of Radiology, University of Minnesota, Minneapolis, MN, United States

**Keywords:** population receptive fields, retinotopy, tonotopy, computational neuroscience, fMRI

## Abstract

In functional MRI (fMRI), population receptive field (pRF) models allow a quantitative description of the response as a function of the features of the stimuli that are relevant for each voxel. The most popular pRF model used in fMRI assumes a Gaussian shape in the features space (e.g., the visual field) reducing the description of the voxel’s pRF to the Gaussian mean (the pRF preferred feature) and standard deviation (the pRF size). The estimation of the pRF mean has been proven to be highly reliable. However, the estimate of the pRF size has been shown not to be consistent within and between subjects. While this issue has been noted experimentally, here we use an optimization theory perspective to describe how the inconsistency in estimating the pRF size is linked to an inherent property of the Gaussian pRF model. When fitting such models, the goodness of fit is less sensitive to variations in the pRF size than to variations in the pRF mean. We also show how the same issue can be considered from a bias-variance perspective. We compare different estimation procedures in terms of the reliability of their estimates using simulated and real fMRI data in the visual (using the Human Connectome Project database) and auditory domain. We show that, the reliability of the estimate of the pRF size can be improved considering a linear combination of those pRF models with similar goodness of fit or a permutation based approach. This increase in reliability of the pRF size estimate does not affect the reliability of the estimate of the pRF mean and the prediction accuracy.

## Introduction

In sensory cortical (and subcortical) areas, processing is organized in maps whose topography depends on the preferences of neuronal populations to the information carried by external stimuli (i.e., the receptive field – RF). Using computational approaches and functional magnetic resonance imaging (fMRI) data, the receptive field of the neuronal population within each voxel (population receptive field, pRF) can be estimated non-invasively at the level of individual subjects. That is, pRF modeling approaches parametrically characterize the measured fMRI response in the space of a chosen set of features that represent the stimuli. Over the last decade, these approaches have become mainstream in neuroscientific fMRI research (Wandell and Winawer, 2015).

Population receptive field modeling has been introduced to study visual information processing building on early techniques for visual field mapping (Engel et al., 1994; Sereno et al., 1995). The pioneering model of Dumoulin and Wandell (Dumoulin and Wandell, 2008) underlies the majority of the current pRF modeling approaches. This model assumes that the pRF characterizing neuronal populations in the visual cortex is a two-dimensional Gaussian function that is defined in the visual space spanned by the stimuli. The Gaussian in the visual field coordinates is described by three parameters, the distance to the fovea (eccentricity), the angle of the pRF mean with respect to the horizontal axis and the standard deviation (pRF size). A number of extensions of this initial model, varying the function that describes the pRF, have been considered. For example the effect of suppressive surround in the visual pRF has been introduced by combining two Gaussian functions (Zuiderbaan et al., 2012; Harvey et al., 2013), and compressive spatial summation has been introduced considering non-linear effects in the pRF model (Kay et al., 2013). Apart from variations on the model shape or properties, different estimation approaches have also been considered. A Bayesian framework for the estimation of the pRF parameters has been introduced allowing model comparison using the posterior probability (Zeidman et al., 2018). Furthermore, using a Bayesian approach, the incorporation of prior anatomical information in the pRF estimation procedure has also been considered (Benson and Winawer, 2018). Another possibility for fitting the Gaussian model is to select the best combination of Gaussian basis functions using LASSO regression. The pRF can also be estimated directly from the features matrix using regularized linear regression, without making *a priori* assumptions about the pRF shape (Moerel et al., 2012; Lee et al., 2013). Apart from characterizing the properties of voxels in response to external stimulation, the pRF modeling approach has also been extended to study resting state connectivity (Gravel et al., 2014). Outside the study of visual areas, pRF modeling approaches have been successfully applied to map the spatial organization of the somatosensory cortex (Puckett et al., 2020) as well as the preference and selectivity (i.e., tuning) to sound frequency in auditory cortical areas (Thomas et al., 2015).

Despite the widespread application of the pRF modeling methodology (Gravel et al., 2014; Zuiderbaan et al., 2017; Dumoulin and Knapen, 2018), there are challenges concerning model fitting that require careful consideration. In fact, finding the best Gaussian pRF representing the responses of an fMRI voxel is a non-convex optimization problem. As a consequence, the cost function (e.g., the minimum squared error or the correlation between the actual voxel response and the response predicted by the pRF model) presents a constellation of local minima and maxima that compromise the convergence of the optimization algorithms conditioned to the signal-to-noise ratio (SNR) of the fMRI data. While this issue has been noted experimentally, here we describe the pRF optimization landscape and show that these local optima appear in a subspace of the cost function space that is populated by very narrow pRFs. This problem is particularly relevant in noisy voxels, where one of the local minima can become the global minimum, and thus result in compromised estimates of the pRF size in particular, but also of the pRF mean. This issue is apparent from the analysis of *in vivo* data where the estimates of pRF size appear to have low consistency both across subjects and within subject (using split-half reliability metrics) (Benson et al., 2018), in contrast with the higher consistency of the pRF eccentricity and polar angle in different areas of the visual cortex. The same issue has been previously highlighted by studies that reported the reliability of pRF parameter estimates in visual cortical areas across sessions (van Dijk et al., 2016) or runs (Zeidman et al., 2018). In addition the estimates of the pRF size have been shown to be more sensitive to changes in the experimental design and the stimuli compared to the estimates of the pRF mean (Alvarez et al., 2015). Different heuristic approaches have been used to mitigate the problem. For example, spatial smoothing can be used to increase the SNR in the fMRI data (Dumoulin and Wandell, 2008) or the analyses can be limited to voxels whose variance explained by the model exceeds a pre-determined threshold (Dumoulin and Wandell, 2008; Thomas et al., 2015). Alternatively, constraints can be introduced to the size of the pRF (i.e., allowing only pRF broader than a certain value) (Zeidman et al., 2018) and a combination of all these approaches has also been considered (Thomas et al., 2015).

These algorithmic alternatives come with multiple software implementations which complicates the validation of the pRF estimation. With the aim of evaluating the reproducibility of these software alternatives a computational framework has been recently developed (Lerma-Usabiaga et al., 2020). Following a similar but complementary aim, this article focuses on the algorithmic principles rather than the software alternatives for estimating the pRF. In particular, we focus on understanding the theoretical difficulties underlying the estimation of the Gaussian pRF model and their interaction with the noise in the signal. To do this, here we first consider simulated data and study the sensitivity of the pRF cost function under variations of the pRF parameters. We focus on the reasons underling the instability (inconsistency) in the estimation of the pRF size. On simulated data that represent the response to traveling bars in the visual field, we compare five pRF estimation methods in terms of bias and variance of the pRF parameters. Four of them are approaches that have been previously introduced. In particular we consider the pRF estimation procedure and an estimation procedure based on grid search extracted from the procedure reported in Benson et al. (2018), the convex estimation procedure introduced by COpRF and the linearized encoding procedure proposed by Lee et al. (2013). The fifth approach consists in a heuristic procedure, that we introduce here, that averages pRF models with similar training error. The advantage of this procedure is a reduction in the variance of the estimated pRF sizes, increasing the reliability of pRFs parameters.

The results obtained in simulations are then validated on real fMRI (visual and auditory fMRI experiments) data by considering the split-half reliability of the pRF parameter. We used the retinotopic experiment from the 7 Tesla Human Connectome Project (HCP) (consisting in 181 subjects – Benson et al., 2018) to evaluate the different methods in their ability of estimating visual pRFs. In this dataset, the Gaussian pRF model is estimated from the response elicited by visual stimuli that smoothly travel across the visual field (see (Senden et al., 2014) for alternatives design that can be used for pRF mapping). In this case the estimation of the pRF occurs in the domain of the fMRI time series by convolving the neural response predicted by the Gaussian pRF model with the hemodynamic response function. However, when the experimental design allows (i.e., when the temporal sequence of the stimuli allows for the estimation of the response to each individual stimulus) the pRF can also be estimated based on a response vector that does not include the effect of the hemodynamic response and constructed by the Beta coefficients estimated with a General Linear Model. In this case, we show that, when the responses to individual stimuli are exchangeable under the null hypothesis, a modification of the grid-search algorithm can be used that leverages a permutation test to estimate the distribution of the cost function under the null hypothesis which is the one of no relationship between the voxel’s pRF model and the observed data. We test this approach on auditory pRFs estimated from the response to natural sounds. In this dataset, selecting the auditory pRF with the smallest probability of occurring by chance results in more reliable estimates than any of the standard methods.

## Materials and Methods

In pRF modeling, the observed fMRI response *y* is assumed to be a function of the pRF (*w*) and a matrix *X* that represents the stimuli (e.g., images or sounds) in the feature space (e.g., pixels or sound frequencies), convolved with the hemodynamic response function:

(1) $$y=\alpha \,{\left(X\,w\right)}^{\gamma }+\varepsilon$$

ε∼N⁢(0,Σ)

The measurement noise is represented by ε which is assumed to be Gaussian with zero mean and covariance matrix Σ. In the visual field the features are measured in degrees of the visual field (Engel et al., 1994) while in the auditory domain the features could represent (logarithmically spaced) frequency bands measured in Hertz (Moerel et al., 2015). The parameter α (usually referred to as pRF gain) adjusts the predicted and observed responses to the same scale. The exponent γ introduces a non-linear relationship between the response and the voxel’s pRF allowing to model compressive summation in the visual field (Kay et al., 2013). The simultaneous estimation of the exponent and the pRF size is hampered by their interaction on the cost function and, for this reason, it is common practice to assume a fixed value for γ (Thomas et al., 2015; Benson et al., 2018). In all subsequent analyses we will use a value of γ = 1 unless otherwise stated. Commonly, the shape of the receptive field is assumed to be an isotropic Gaussian *w*(*r*|μ,σ), which is a function of two parameters, the pRF mean μ and the pRF size (standard deviation σ).

(2) $$w_{i}=e^{-\frac{{\left(r_{i}-\mu \right)}^{T}\,\left(r_{i}-\mu \right)}{2\,\sigma^{2}}}$$

In the visual domain, this function describes the response of a voxel to a stimulus presented at position *r*_**i**_ in the feature space (Dumoulin and Wandell, 2008). In the auditory domain *r* typically refer to spectral characteristics of the sounds. Note that in Eq. 2 the pRF is described by a multidimensional Gaussian (*r* and μ are vectors) whereas the intensity of the response at a particular location is a scalar value resulting from the multiplication between the matrix *X* and the vector *w*.

### Real Data

#### The HCP Dataset

The retinotopy data from the Young Adult Human Connectome Project (HCP) contains pre-processed fMRI data from 181 subjects acquired at 7T with isotropic voxels of 1.6 mm. The dataset and the codes for retinotopic analysis are available from^1^ and^2^ respectively. In this dataset, after preprocessing, the voxel size was resampled to 2 mm isotropic and projected on the cortical surface. The experimental design consisted of six fMRI runs of 300 s each acquired with a TR of one second, in which visual stimuli (bars) where presented traveling smoothly across the visual field. To evaluate the reliability of the pRF parameter estimates we have split each of the runs in two halves (150 fMRI volumes each). Following a previous report (Benson et al., 2018), we analyzed the data coming from four regions of interest in visual cortex defined as posterior (V1-V3), dorsal (V3A/B, IPS0-5), lateral (LO-1/2, TO-1/2), and ventral (VO-1/2, PHC-1/2), with a total of 5012 voxels in each subject. A detailed description of the experiment can be found in Benson et al. (2018). Due to its large number of subjects and the standardization of the preprocessing pipeline this dataset is an excellent benchmark sample for a wide range of applications, including the validation of pRF methods.

#### Natural Sounds Experiment

Subjects with no history of neurological disease took part in the experiment and gave informed consent before commencement of the measurements. The Ethical Committee of the Faculty for Psychology and Neuroscience at Maastricht University granted approval for the study. Magnetic resonance imaging data were acquired on an actively shielded MAGNETOM 7T whole body system driven by a Siemens console at Scannexus https://scannexus.nl/. Functional (T2^∗^ weighted) data (1.1 mm isotropic) were acquired using a clustered Echo Planar Imaging (EPI) technique. The experiments were designed according to a fast event-related scheme. A total of 168 natural sounds were presented six times across 24 runs in silent gaps in between volume acquisitions using magnetic compatible earbuds (Sensimetrics inc.). The 168 sounds were divided into four training and testing sets (126 and 42 sounds respectively) without overlapping sounds between the training and test set. For more details on the acquisition and stimulation paradigm we refer to (Sitek et al., 2019). We analyzed data in the original volume space (without projection on the cortical surface) confined to an anatomical mask covering the auditory cortex (Heschl’s gyrus, planum temporale and planum polare) bilaterally. We estimated pRFs from the voxels’ beta time series (i.e., the estimated response to all individual natural sounds in the experiment). Beta series estimation followed the procedure detailed in Sitek et al. (2019), the codes are publicly available at https://github.com/sitek/subcortical-auditory-atlas. Only voxels that belonged to the top 10% of the distribution of F-values estimated on the training data were submitted to the pRF analysis. On average this selection criterion produced a set of 20000 voxels in the sample of 10 subjects. Note this number is approximately five times larger than the number of voxels that were submitted to the pRF analysis in the visual dataset example. The unprocessed fMRI time series for the 10 subjects can be downloaded from https://openneuro.org/datasets/ds001942/versions/1.2.0. To estimate a Gaussian pRF in the sound frequency space, natural sounds were analyzed in order to extract the norm (energy) of the Fourier transform at 2048 logarithmically scale frequency partitions from 180 Hz to 7040 Hz.

### Simulations

We used simulations to compare pRF estimation procedures. The first simulation corresponds to a one dimensional pRF characterizing the preference to sound frequencies that can be estimated in auditory fMRI experiments. We used this simulation to introduce the optimization landscape typical of pRF modeling. The second simulation considers the case of a two dimensional pRF representing the retinotopic preference that can be estimated from visual fMRI experiments. We used this simulation both for introducing issues relating to the optimization of the pRF paramters as well as for a comparison between estimation approaches.

To introduce the optimization landscape and provide a simple graphical representation, we started with simulating a one-dimensional pRF, modeling the response of a voxel to 240 equally spaced tones between 88–8000 Hz presented randomly in one single fMRI run. This simulation followed the experimental design adopted by Thomas et al. (2015), in which one fMRI run contains 260 volumes (TR = 2000 ms). We simulated the response elicited by a pRF with mean and size defined by [μ_0_ = 120 (4.04*KHz*),σ_0_ = 16.2 (534*Hz*)] and a scaling parameter of α = 1. To the simulated fMRI response (obtained following Eq. 1) we added realistic fMRI noise generated with a first order autoregressive processes with an autoregressive coefficient of 0.36 (implemented using the Matlab function arima.m) (Worsley and Friston, 1995). The standard deviation of the noise defines the SNR, and it affects the maximum performance that a model can attain in predicting fMRI responses. In reporting the results of our simulations we quantified the noise level by computing the split-half noise ceiling (SHnc) between two signals generated under the same simulation framework (Lage-Castellanos et al., 2019). We used the SHnc as to quantify signal to noise ratio as it relates to the reproducibility of the data and is an estimator of the maximum prediction accuracy that a pRF model can attain given the noise in the data. As we have previously shown this is a robust estimate that does not rely on specific assumptions with respect to the noise structure (Lage-Castellanos et al., 2019). We used this simulated data to analyze the effect of the noise on the estimation of the pRF parameters in two representative scenarios with high and low noise ceiling (i.e., SHnc values of 0.63 and 0.11 respectively).

**FIGURE 1:**
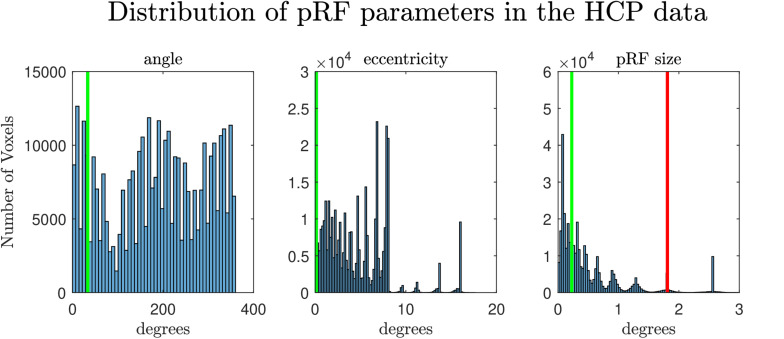
Distribution of the estimated pRF parameters (angle, eccentricity and pRF size) from the retinotopy experiment of the HCP database (Benson et al., 2018). The vertical lines denote the values of the parameters of the pRF used in the second simulation. In the extreme right the green and the red lines denote the pRF sizes used for simulating the narrow and the broad pRFs respectively.

In a second set of simulations, we considered a two-dimensional pRF, modeling the responses of a voxel in visual cortex to retinotopic mapping stimuli. We followed the experimental design of the retinotopic experiment of Human Connectome Project (see e.g., Benson et al., 2018). We simulated the response of two voxels with different pRFs (eccentricity: 1.79°; angle: 0.59 radians, 33.8°), one narrow and one broad (size: 0.23° and 1.81°, respectively). In Figure 1 we present the values of the simulated pRF parameters in the second simulated dataset in the context of the pRF parameters estimated from the HCP data. To evaluate the effect of SNR we considered three scenarios with different noise ceilings: high (0.6 SHnc), medium (0.35 SHnc), and low (0.1 SHnc). These noise levels match the distribution of the SHnc in the real data used in this article (see Supplementary Figure S1). In order to evaluate the bias and variance of the estimated pRF parameters by different procedures, the second set of simulations was repeated 1000 times.

### pRF Model Estimation

To estimate the pRF, most applications use as cost function either the sum of squared error (C_SSE_) or one minus the correlation (C_1−corr_). In the case of a linear model (i.e., γ = 1), these cost functions are defined as:

(3) $${\text{C}}_{\text{SSE}}={\left(y-\alpha \,X\,w\,\left(\mu ,\sigma \right)\right)}^{\text{T}}\,\left(y-\alpha \,X\,w\,\left(\mu ,\sigma \right)\right)$$

$${\text{C}}_{1-\text{corr}}=1-\frac{y^{\text{T}}\,X\,w\,\left(\mu ,\sigma \right)}{\sqrt{w^{T}\,X^{T}\,X\,w}}$$

$$\alpha^{*},\mu^{*},\sigma^{2,*}={\text{argmin}}_{\alpha ,\mu ,\sigma }^{2}\,C\,\left(\alpha ,\mu ,\sigma^{2}\right)$$

Here, without loss of generality, we assumed *y* and the columns of **X** to have zero mean, which makes the definition of C_1−corr_ simpler and have omitted the variance of *y* in the definition of C_1−corr_ since it is not a function of the pRF parameters and only acts as a scaling parameter for the cost function of each voxel. If the variance of *y* is not unitary the value of C_1−corr_ is a linear function of one minus the correlation coefficient and is not bounded in the interval [0 2]. Note that the minimum of the cost function is invariant to this linear transformation.

In the original (Dumoulin and Wandell, 2008) procedure, the pRF parameters(μ,σ) are optimized in a two-step approach using C_SSE_ as a cost function. The scaling factor (α) was linearly solved to account for the unknown units of the fMRI signal. For $\alpha ={\left(w^{T}\,X^{T}\,X\,w\right)}^{-\frac{1}{2}}$ both cost functions C_SSE_ and C_1−corr_ are equivalent (see Supplementary Material 1.1), but not equivalent to the original procedure in which α is used to adjust the scale of *y* (Dumoulin and Wandell, 2008). Note that for C_1−corr_ the scale of *y* is not relevant. Trying to optimize μ, σ and α at the same time increases the number of local extrema since varying either α or σ have similar effect on the cost function, and results in imprecise estimations for α and/or σ (Zeidman et al., 2018). Finally, minimizing C_1−corr_ is equivalent to maximizing the correlation coefficient between the predicted and observed data (C_corr_) and in this manuscript when presenting the results of our analyses we will use C_corr_.

**FIGURE 2:**
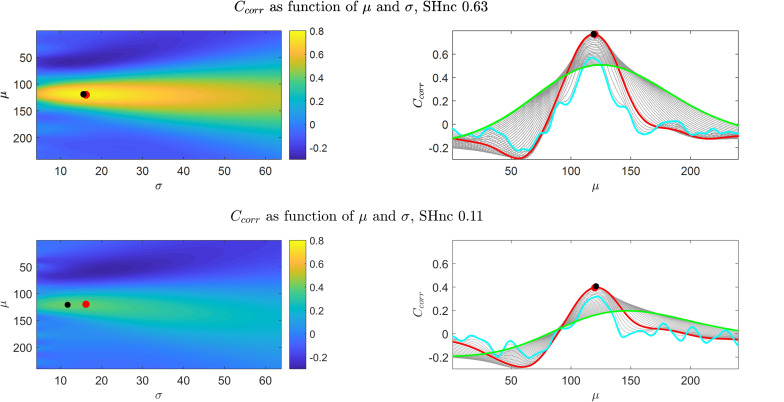
Results obtained from the simulations of the one dimensional pRF characterizing the preference to sound frequencies (i.e., auditory fMRI data). Upper and bottom **left panels** show C_corr_ as a function of μ and σ for high and low SHnc respectively. The **right panels** show the corresponding profiles with respect to μ, while the different (gray) curves are obtained with different σ values. The values of C_corr_ as a function of μ for the broadest and narrowest possible pRFs are displayed in green and cyan respectively. The values of C_corr_ as a function of μ for the pRF that generated the data are depicted in red. Red dots and black dots denote the μ,σ used to generate the data and the global minimum respectively. In the high SHnc scenario the generative pRF (red) and the global minimum (black) are in the same location in the (μ,σ) space (the dots are superimposed).

Figure 2 (left panels) shows for the first simulation (auditory data experimental design) the value of the cost function (C_corr_) for every possible combination of the pRF mean (μ - scalar for the one dimensional Gaussian simulation) and size (σ) obtained with high (top) and low (bottom) noise ceiling. The right panels of Figure 2 show the dependence of the cost function C_corr_ on μ, with different curves corresponding to different σ values (depicted in gray). The results for C_SSE_ are presented in the Supplementary Figure S2. Both when using the correlation or the residual sum of squares, the cost function varies little along the σ axis in comparison to the μ axis (note the left panels of Figure 2 and Supplementary Figure S2), and, as a result, is more sensitive to variations in the pRF mean than in the pRF size. An interesting observation can be made when comparing the right panels of Figure 2 and Supplementary Figure S2. When considering the behavior across a large range of possible pRF means, the C_corr_ of narrow models (cyan line) is generally worse than the one of broad models (i.e., the cyan line is above the green one and C_corr_ is better when is higher). This behavior is opposite for C_SSE_. Importantly, around the optimal pRF mean narrow and broad models result in a similar behavior when using both C_corr_ and C_SSE_. In what follows for simplicity we will present results obtained using the correlation as cost function, but the conclusions we take apply to the use of the sum of square errors given that both cost functions present the same behavior with respect to changes in the pRF size. In Supplementary Figure S3 we present the ratio between the derivatives of the cost function (C_corr_) with respect to μ and σ for two different noise levels as it allows to visualize the regions of the optimization landscape in which either of the parameters (the pRF mean or size) have more influence on the cost function. The formulas for the derivatives with respect to μ and σ are presented in the Supplementary Material 1.2. It is important to note that, in low noise ceiling conditions (bottom in Figure 2 and Supplementary Figure S2), the global minimum of the cost function shifts away from the true pRF mean and sigma and local extrema emerge compromising the convergence of pRF estimation. The number of local extrema (with respect to μ) increases with decreasing values of σ (narrowest pRF, cyan curve in the bottom right panel of Figure 2). In Supplementary Material 1.3 we show that when σ→0 the derivative of the cost function tends to zero. That is, for σ→0 the cost function corresponds to either a local minimum or a local maximum. As a result the minimization of the cost function in noisy voxels may be biased toward narrow pRFs, and to the limit the pRFs can result in selecting a single feature in the stimulus space. In particular high frequency noise with sharp spikes can bias the estimation in the direction of narrow pRFs as the prediction of a pRF selecting a single feature in stimulus space result in a prediction with sharp peaks (every time this feature is stimulated).

**FIGURE 3:**
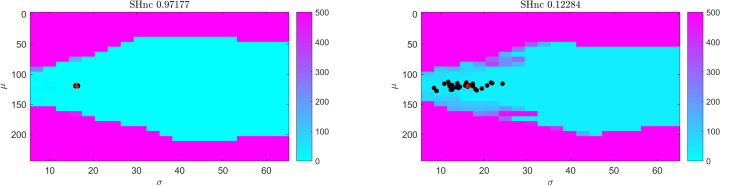
Results of the convergence of the optimization of a one simulated dimensional pRF characterizing the preference to sound frequencies (i.e., auditory fMRI data; high **(left)** and low **(right)** split-half noise ceiling). Colors indicate the distance (squared Euclidean norm) between true parameter values (red dot) and the solution obtained from starting Levenberg-Marquardt optimization at each location in parameter space. Distance values are averaged over 30 realizations. Black dots indicate the initial conditions (for pRF estimation using LMA) estimated from grid-search. In particular a cyan color indicates the starting values for pRF mean and size that resulted in the Levenberg-Marquardt algorithm to converge to the simulated value.

For gradient-based optimization methods, whether the algorithm asymptotically approaches the global minimum as the iterations proceed (i.e., convergence) crucially depends on the initial conditions. In Figure 3 we show the distance (measured as squared Euclidean norm) between simulated pRF parameter values (μ and σ defined by the red dot in correspondence with Figure 2, auditory data) and the estimates obtained from the Levenberg-Marquardt algorithm (LMA) as a function of the initial conditions averaged over 30 realizations of the noise (high and low SHnc in left and right panels of Figure 3 respectively). We used here C_corr_ as cost function and the Levenberg-Marquardt algorithm for optimization. The pRF size was constrained to be a positive number. The same results were obtained when the estimation was carried on using gradient-descend (data not shown). The turquoise (pink) region indicates for which initial conditions the estimation procedure converges to the global minimum. In line with the results reported in Figure 2 indicating that the number of local minima depends on the pRF size, the region of convergence narrows as σ decreases. This renders the choice of a large sigma as initial condition safe irrespective of the true size of the receptive field. Typically, initial conditions for pRF parameter estimation are obtained from grid-search. In our simulation the initial conditions obtained from grid-search (black dots in Figure 3) are always falling within the region of convergence for the subsequent LMA optimization even at the highest level of simulated noise.

**FIGURE 4:**
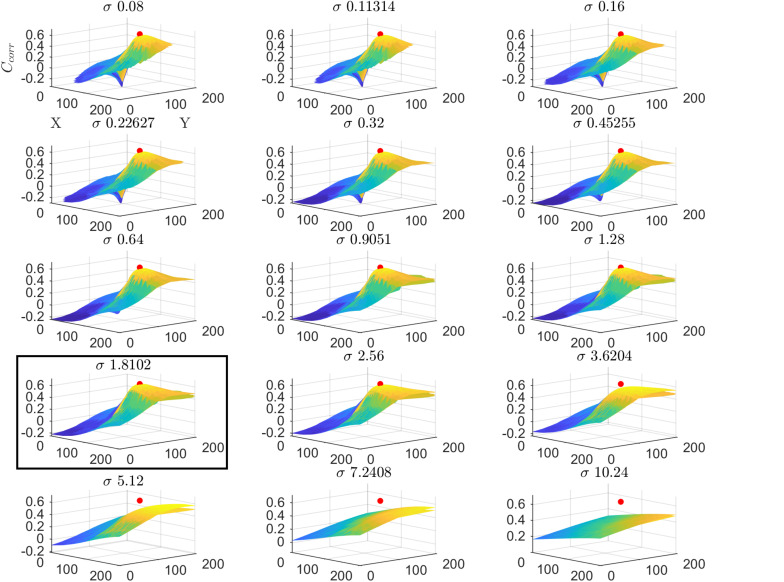
Cost function (C_corr_) in the space of the pRF paramters (x,y,σ) for a voxel from the HCP dataset in the first split of the data (subject code 100610, first split, voxel index 5). The location of the global maximum is represented by a red dot and is obtained σ = 1.81. Each subplot represents the cost function at varying values of μ (defined by (x,y)) while the different subplots are obtained varying the value of the pRF size (σ). For visualization clarity in this figure we used cartesian coordinates instead of eccentricity and angle.

**FIGURE 5:**
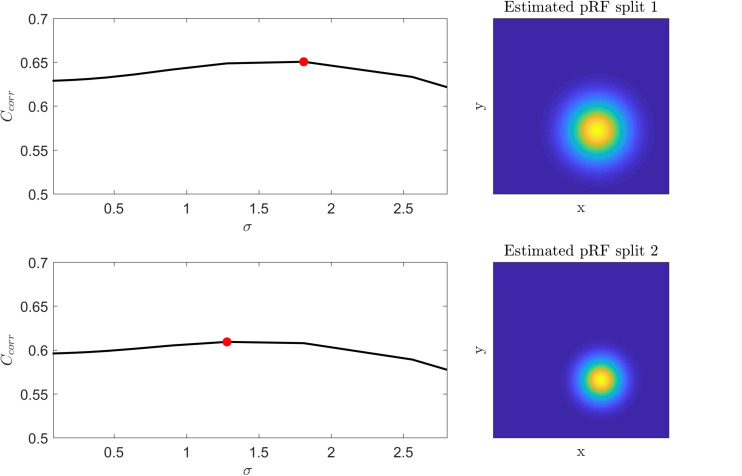
**Left:** Cost function (correlation coefficient) as a function of the pRF size (σ) for the two splits of a voxel in the HCP dataset. The cost function was computed by fixing the voxel’s pRF mean (x,y) to the value of the best fit model that corresponds to the global maximum (red dot in the figure). **Right:** The corresponding estimated pRFs for each split.

We tested the insights on the behavior of the optimization method gathered on simulated data on real fMRI data. Figure 4 displays C_corr_ for different values of μ [defined by (x,y)] and σ from a voxel in the HCP dataset. Note that, consistently with the simulations presented in Figure 2, the cost function varies mainly with respect to μ (for a fixed value of σ ; i.e., within each plot) while the variability between plots (i.e., for different σ) is smaller. Figure 5 presents the variation of C_corr_ with respect to σ for the optimal pRF position (μ) for the two independent data splits in real fMRI data (same voxel from the HCP data set presented in Figure 4). For a range of σ between [0 – 2.5] the cost function is constrained in the small interval of [0.63 – 0.65] and [0.59 – 0.61] (correlation coefficient) for the first and second splits respectively. The global optimum occurs at locations σ_1_ = 1.81 and σ_2_ = 1.28 that is a change in width of the pRF across the two splits of 1.4 times. The corresponding estimated pRFs are displayed in the right panels of Figure 5. The ratio between σ_1_ and σ_2_ for all voxels in the visual cortex (subject code 100610) is presented in the Supplementary Figure S4.

These analyses in both simulated and real data illustrate the low sensitivity of the cost function (both the correlation and the residual sum of squares) to variations in σ resulting in the large variability in the estimated pRF size that has been previously reported. We show that this problem is amplified by the noise in the fMRI data and that the variability in the estimate of the pRF size is contrasted by the relative robustness of the estimate of the pRF mean.

### pRF Estimation Methods

In this section we briefly describe the algorithmic principles used by different pRF estimation methods. Note that different software implementations of the same algorithm are available and they may differ in computational efficiency, number of hyperparameters and programming language. For an exhaustive comparison between software tools for estimating the pRF see (Lerma-Usabiaga et al., 2020).

#### Two-Steps Optimization [GridSearch-Gradient Based] (HCP7pRF)

Here we used the implementation of the method presented in Benson et al. (2018) which is available at https://osf.io/bw9ec/. Following the approach of Dumoulin and Wandell (2008) this method estimates the pRFs in two-steps. A grid-search is used for selecting initial values for the pRF parameters for subsequent non-linear optimization. The grid search optimization uses the correlation coefficient as the cost function. The initial conditions are selected within a dictionary of plausible pRFs. In this dictionary the angles of all possible pRFs are uniformly distributed between [0° and 360°]. On the other hand, eccentricity and pRF size are characterized by non-uniform distributions with the density of pRFs decreasing with eccentricity and more seeds characterized by a small σ (i.e., narrow pRF). The scaling parameter α is computed using linear regression between the observed and predicted fMRI time series (the latter obtained after selecting the best seed with grid search optimization). The pRF parameters obtained in the first pass (using grid search) are then submitted to a second non-linear optimization pass using the Levenberg-Marquardt algorithm implemented by the Matlab function lsqcurvefit. This second optimization pass uses the C_SSE_ as the cost function. In this approach, the observed voxel’s fMRI time series is standardized to have zero mean and unitary variance. The non-linear optimizer also allows for the eventual optimization of the compressive non-linearity (γ) (Kay et al., 2013). Here, we used default setting for all functions in this toolbox.

#### Two-Steps Optimization [GridSearch-Gradient Based] for Auditory Data (GS + Gb)

A two-steps optimization approach has also been developed to estimate auditory pRFs (Thomas et al., 2015). Here when analyzing auditory fMRI (or the simulated auditory responses) we used this approach whose implementation is available at https://github.com/kellychang4/pRF. This approach, firsts uses grid-search to obtain an estimate of the initial conditions to be refined in a second step. The dictionary used in the grid-search considers pRFs with logarithmically spaced (tonotopic) preferences (i.e., the mean of the pRFs) between 180 Hz and 7040 Hz, while the pRF size varies linearly from 10 to 100 in full width at half-maximum (FWHM) of the logarithmic spaced bins. After the grid-search, the estimation of the model is refined using the Nelder-Mead algorithm (Lagarias et al., 1998 implemented by the fminsearch Matlab function) which is a direct search method and does not rely on the gradient of the cost function. It is important to note that in this procedure both the grid search and the finer optimization step are carried on using C_corr_ as the cost function (which is a difference with respect to the two-step procedure outlined above for the analysis of visual data which uses C_SSE_ in the second optimization step).

#### Grid-Search (GS)

Grid-search automatically imposes a form of regularization on the models avoiding the optimization to converge into to the subset of the local minima not included in the grid. For this reason, we included here the estimation of pRF using only grid-search and used, for the auditory and visual data, the grid-search module from the HCP7pRF procedure. The regularization is given by the coarseness of the grid. A coarse grid will reduce the variability of the estimated pRF while paying the cost of introducing an estimation bias. A very fine grid will produce unbiased estimates however increasing the risk of landing in local minima. Consistently with the implementation of grids search available in the two-step optimization procedures for both visual and auditory data (see above) we used C_corr_ as the cost function.

#### Convex pRF Estimation (COpRF)

We used the implementation available at https://github.com/davesl/COpRF. The algorithm estimates the voxel’s pRF as a linear combination of the pRF seeds available in a large pRF dictionary using LASSO regression (Bishop, 2006). The Lasso regression minimizes C_SSE_ where the voxel’s fMRI time series and the pRF dictionary have been standardized to have zero mean and unitary norm. As Lasso imposes sparsity on the regression coefficients only a small subset of the pRF dictionary will be selected for obtaining the voxel’s pRF. Interestingly, two consecutive regressions are performed using different dictionary sizes. The first regression uses a reduced dictionary of around 3000 seeds. Then the seeds from the large dictionary that are contained within a radius from the center of the pRF estimated in the first pass are submitted into the second regression. It is important to note that estimated pRF parameters for the voxel’s pRF are derived by the weighted sum of the pRF parameters of the selected seeds using the LASSO coefficients. Here we used the default values in the toolbox for the search radius and the LASSO regularization parameter.

#### Linearized Encoding (LinEnc)

We used the implementation available at: https://sites.google.com/site/leesangkyun/prf/codes.zip, (Lee et al., 2013). Linearized encoding models do not assume a specific shape for the pRF, instead using the General Linear Model (GLM) the (linear) contribution of each model feature to the fMRI time series (or Beta series) is estimated using ridge regression (Bishop, 2006) which uses C_SSE_ on the space of the original variables (untransformed). Regularization is required as the number of model features (e.g., points in the visual field, or frequencies of a sound) is often larger than the number of time points in the fMRI time series (or the number of stimuli that define the length of the Beta series). In the original settings of this toolbox the voxel’s pRF is estimated only if the explained variance is larger than 0.3 after ridge regression. Here, to compare all methods under the same conditions, we performed the estimation for all voxels. For the estimation of visual pRFs we down sampled the visual stimuli to a square of 101 × 101 pixels. We used the default parameters of the toolbox (i.e., the grid of regularization parameters (λ) to be used in the ridge regression). To estimate a preferred feature and the size of the pRF, in a second step a Gaussian function is fit (using the Matlab function lsqcurvefit) to the estimated linear coefficients after imposing a threshold for discarding noisy coefficients. We used the default setting for this second step by considering three different thresholds (0.3, 0.5, and 0.7; relative to the value of maximum coefficient) and selecting the Gaussian model that explained the highest amount of variance of the linear coefficients obtained from the first step.

### Modifications to the Grid Search Procedure

In this section we describe the two variants of the grid search algorithm. We propose these modifications with the purpose of increasing the reliability of the estimate of the pRF size. The first approach combines the pRF models in the search space that result in similar goodness of fit to the data. The second modifies the criterion for selecting the best pRF model. In particular, instead of selecting the pRF model that maximizes the correlation to the observed data, we select the model with minimum probability of being obtained under the null hypothesis of no relationship between the observed and predicted responses.

#### Averaging pRF Models Explored With Grid-Search (ModelAve)

To reduce the variability of the pRF estimates, we propose to average pRFs models obtained from a grid-search and that have similar goodness of fit using C_corr_ as the cost function. We used the grid-search module of the two-step optimization procedure. However, instead of selecting the pRF model that produces the maximum correlation with the observed response (as in the grid-search procedure described above), we propose to consider all pRFs models within a range of 1% of the goodness of fit of the best model. The voxel’s pRF can then be obtained as the average of the pRF models in this interval. Note that the average is performed in the pRF space instead of the parameters space (as done by the COpRF procedure). Despite the resulting averaged pRF not being necessarily a Gaussian function, to estimate pRF parameters (μ and σ) we fitted a Gaussian to the averaged pRF. This approach can be used both on the original fMRI time series (for experimental designs such as the traveling bars in the visual field that do not allow the estimation of single stimuli) as well as on Beta time series. Here we used this approach to analyze both visual and auditory fMRI data.

#### Permutation Based Model Grid-Search for Separable Betas Design (PermGS)

This procedure can only be implemented when the Beta responses are exchangeable under the null hypothesis. The experimental design of the retinotopy HCP dataset consists of visual stimuli that smoothly travels across the visual field. This experimental design prevents the estimation of separate betas associated with each stimulus and, as a consequence, the permutation procedure cannot be used. For this reason, here we used this method only when analyzing the auditory data. The distribution of local minima in the optimization space that we have highlighted in simulations (Figures 2–4) results in a large probability for the best model to be characterized by small σ just by chance. Such probability can be estimated by empirically determining the null distribution of the cost function (C_corr_) for every possible combination of pRF mean and size using permutations. The permutation procedure consists in randomizing the response vector (here the beta series estimated from the fMRI time series as the response to all the stimuli) while keeping the pRF model fixed. By defining the empirical null distribution we selected, for every voxel, the pRF model (i.e., selecting the pRF mean and size) with the smallest probability of occurring by chance.

### Statistical Analysis

The results obtained on simulated data were statistically assessed using bootstrapping in order to evaluate the bias of the pRF estimates as well as for comparing the variance of the estimates across methods. In particular, the distributions of pRF parameters (angle, eccentricity and size in the case of the simulated visual data) obtained from the 1000 simulations were bootstrapped 10000 times (i.e., we derived 10000 bootstrap samples each one representing the distribution of a given pRF parameter over 1000 simulations). To statistically evaluate the bias in the estimated parameters we computed the α = 0.05 (two tailed) confidence intervals of the mean of each bootstrap sample. The bootstrapping procedure for the pRF was implemented using R and a package (circular) allowing to account for circular data^3^. When the value of the pRF parameter used for simulating the data (e.g., the simulated parameter) fell outside the confidence intervals we concluded that the method is biased and estimated the bias as the distance between the simulated parameter and the mean across all bootstrap samples. When a significant bias was observed, we reported the effect in terms of the original units (e.g., degrees of the visual field). To compare the variability of the estimates (for each pRF parameter), we computed the ratio of the variance between bootstrap samples (paired) obtained using different approaches. This results in 10000 values of the ratio between two methods. Estimating the α = 0.05 confidence interval of this distribution allowed us to evaluate if there were significant differences between methods (i.e., when the value 1 indicating identical variability between two methods was not included within the confidence interval).

The statistical analyses of the estimates obtained on the HCP dataset were performed using bootstrapping. In particular, we bootstrapped 10000 times the 181 values of reliability (one value per subject) obtained by correlating the estimates of each of the pRF parameters obtained across the two splits of the data. By estimating confidence intervals (α = 0.05) for the difference between each pair of methods we determined the significance of the differences and report the effect size as the mean differences in reliability between methods. We followed a similar approach to compare prediction accuracy between methods.

For the auditory data, we tested for statistical differences (in reliability of the estimates measured as the Spearman correlation between independent estimates of the pRF parameters) using permutation tests. The empirical null distribution of the difference in reliability between each pair of methods was obtained by exchanging the values between methods. The p-values (threshold at α = 0.05, two tailed) were computed as the cumulative probability of obtaining a reliability greater or equal (smaller or equal for the left tail) than the observed difference under the distribution of the null hypothesis.

## Results

The different estimation methods use different cost functions (some the correlation and some the sum of squares). Our previous analyses showed that the sensitivity of these cost functions to variations in the pRF size (and mean) is similar (see Figure 2 and Supplementary Figure S2) and in Supplementary Material (Supplementary Figure S5) we present the results on simulated data in which we changed the cost function for the Grid Search from the correlation to the sum of squares. These results show that the choice of the cost function does not influence our evaluation on the reliability of the estimates of the pRF size.

### Results for Simulated Visual Data

**FIGURE 6:**
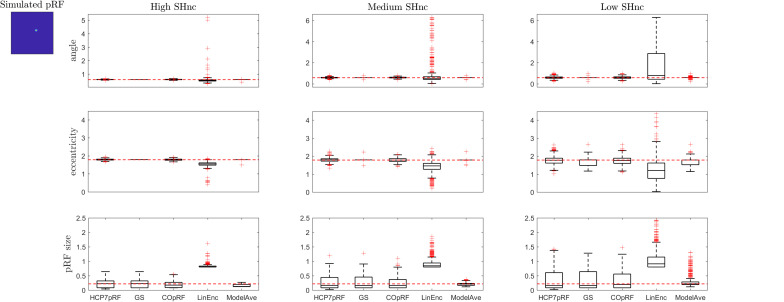
Boxplots ([25, 50, 75] percentiles over 1000 simulated datasets) represents the estimated pRF parameters using different estimation procedures for a narrow two-dimensional pRF (presented in extreme left). Each column refers to a different split-half noise ceiling level from high to low: High SHnc = 0.63, Medium SHnc = 0.35, Low SHnc = 0.1. Each row presents the results for each of the pRF parameters: angle, eccentricity and pRF size. Outliers (in the estimated pRF parameters) are denoted by red + symbols and defined by the condition of being outside the 1.5 interquartile range. The horizontal red line indicates the pRF parameters used to simulate the data.

**FIGURE 7:**
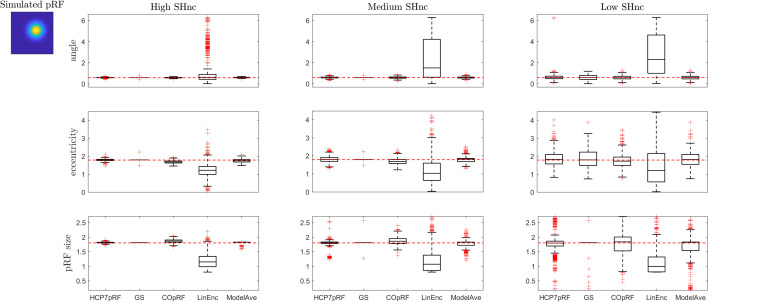
Boxplots ([25, 50, 75] percentiles over 1000 simulated datasets) represents the estimated pRF parameters using different estimation procedures for a broad two-dimensional pRF (presented in extreme left). Each column refers to a different split-half noise ceiling level from high to low: High SHnc = 0.63, Medium SHnc = 0.35, Low SHnc = 0.1. Each row presents the results for each of the pRF parameters: angle, eccentricity and pRF size. Outliers (in the estimated pRF parameters) are denoted by red + symbols and defined by the condition of being outside the 1.5 interquartile range. The horizontal red line indicates the pRF parameters used to simulate the data.

Figures 6, 7 report the comparison between the tested pRF estimation procedures in terms of bias and variance of the estimates on the second simulated dataset (i.e., two visual pRFs, one narrow and one broad) at three levels of noise (high, medium, and low).

For a narrow pRF (Figure 6 and Table 1), COpRF and ModelAve showed biased estimates of the eccentricity and the pRF size. LinEnc produced biased estimates as well, but, in particular for eccentricity and pRF size the bias was larger in effect size compared to the bias introduced by ModelAve and COpRF (at all considered noise levels). We did not detect a significance bias for HCP7pRF and grid-search. However, HCP7pRF is two orders of magnitude more computationally demanding than grid-search (see Supplementary Table S1). This pattern was largely confirmed when considering large pRFs (Figure 7 and Supplementary Table S2). In this case, we did not detect a significance bias for grid-search (all parameters). LinEnc produced biased estimates of the eccentricity and pRF size which were larger in effect size compared to the bias introduced by ModelAve and COpRF (at all considered noise levels). We compared the variability of the estimates of the pRF between pairs of methods by statistically assessing (with bootstrapping – see section “MATERIALS AND METHODS”) the ratio between the variance of the estimates. The results of this statistical analysis are reported in Table 2 for a narrow pRF and Supplementary Table 3 for a broad pRF. Averaging models with similar goodness of fit (ModelAve) prior to the estimation of the pRF size resulted in a smaller variability of the estimates at all the considered noise levels for narrow pRFs (Table 2). When considering broad pRFs (Supplementary Table S3) the advantage of using model averaging with respect to the variability of the estimates was less noticeable. Note that reducing the variability of the pRF size estimates for narrow pRFs will have a large impact on the analysis of real data, as the largest proportion of voxels in visual cortex present narrow pRFs (see Figure 1). Linearized encoding showed the larger bias in the estimate of the pRF parameters (eccentricity and size) for all considered noise levels and this bias did not correspond to a significant gain in variance reduction when comparing its estimates to e.g., model averaging.

**TABLE 1:** Statistical analysis of the bias of the estimates of the angle, eccentricity and pRF size obtained when simulating a narrow visual pRF (angle = 0.59 radians, eccentricity = 1.79°, pRF size σ = 0.23°) at different noise levels.

	**Low Noise**			**Medium Noise**			**High Noise**		
	**Angle**	**Ecc.**	**Size**	**Angle**	**Ecc.**	**Size**	**Angle**	**Ecc.**	**Size**
HCP7pRF	n.s.	n.s.	n.s.	n.s.	n.s.	n.s.	n.s.	n.s.	n.s.
GS	n.s.	n.s.	n.s.	n.s.	n.s.	n.s.	n.s.	n.s.	n.s.
COpRF	n.s.	−0.007	−0.05	n.s.	−0.01	−0.06	n.s.	−0.03	n.s.
LinEnc	n.s.	−0.24	0.57	n.s.	−0.33	0.61	n.s.	−0.59	0.68
ModelAve	n.s.	−0.006	−0.09	n.s.	−0.006	−0.01	n.s.	−0.006	n.s.

*For every noise level, columns represent the different parameters while rows represent the different estimation methods. For significant effects (*p* < 0.05) we report the effect size (in units of the parameter), while n.s. indicates a non-significant effect. When significant bias was observed, the corresponding cell displays the mean of the estimated parameter minus the its true value.*

**TABLE 2:** Pairwise comparison of the variability of the estimates of the pRF size between estimation methods at different levels of noise (narrow pRF: angle = 0.59 radians, eccentricity = 1.79°, pRF size σ = 0.23°).

	**Low Noise**				**Medium Noise**				**High Noise**			
	***GS***	***COpRF***	***LinEnc***	***ModelAve***	***GS***	***COpRF***	***LinEnc***	***ModelAve***	***GS***	***COpRF***	***LinEnc***	***ModelAve***
HCP7pRF	n.s.	1.24	5.56	9.10	n.s.	1.12	1.67	12.53	n.s.	1.13	n.s.	3.93
GS		1.24	5.61	9.16		1.12	1.68	12.62		1.14	n.s.	3.87
COpRF			4.48	7.33			1.48	11.17			n.s.	3.47
LinEnc				n.s.				7.45				3.84

*Every cell displays (for significant effects) the variance of the pRF size estimated by the method in the row divided by the variance of the method in the column ($\frac{\mathrm{Var}\,{\left(\sigma \right)}_{\mathrm{row}}}{\mathrm{Var}\,{\left(\sigma \right)}_{\mathrm{col}}}$), while n.s. indicates a non-significant effect.*

**FIGURE 8:**
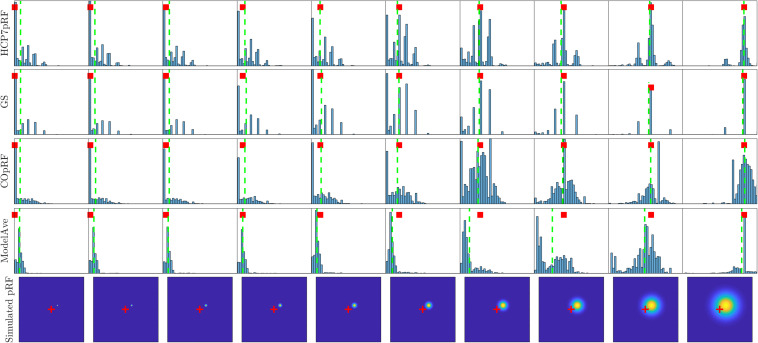
Histograms of the estimated pRF size for HCP7pRF, GS, COpRF and ModelAve. The bottom row shows the simulated pRF (the fovea is marked in red). In all other plots, the red square denotes the pRF size value used to simulate the data (i.e., the targeted value to be estimated). The vertical green lines denote the median of the distribution of the estimated pRF size.

A more refined evaluation of the estimated pRF size for monotonically increasing simulated pRF sizes and moderate noise level (SHnc = 0.35) is presented in Figure 8. Each simulation was repeated 1000 times and the empirical distribution of the estimated pRF size is presented for HCP7pRF, GS, COpRF and ModelAve. The LinEnc algorithm was discarded as this method showed the largest bias and not significantly improved variability of the estimates when comparing it to model averaging (see Tables 1, 2 and Supplementary Tables S2, S3). Our results indicate that, within the range of the pRF size we considered here, combining models with similar performances (ModelAve) reduced the variance of the estimates for pRF size smaller than σ = 0.91 (see Supplementary Table S4). While model averaging resulted in a reduction in variance, for a large range of pRF sizes, it was also associated with an increased bias of the estimated of the pRF size as evidenced by the separation between the median (green lines in Figure 8) and the value of the pRF size used for simulating the data (red square).

Computational times for all tested approaches relative to GS are presented in Supplementary Table S1 for the estimation of a narrow (visual) pRF (for a varying number of simulated voxels), and at a medium noise level (SHnc = 0.35). The results indicate that the two step optimization procedure (HCP7pRF) is the most computationally demanding approach (at least two orders of magnitude slower than GS).

### Visual pRFs Estimated From the Human Connectome Dataset

**FIGURE 9:**
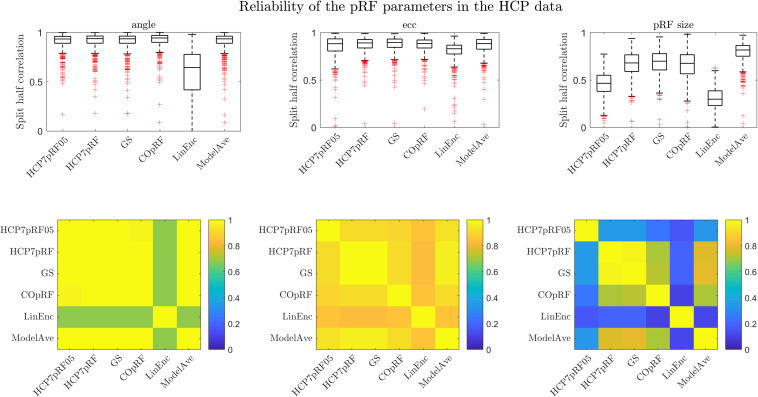
The split-half reliability ([25, 50, 75] percentiles of the distribution across 181 subjects) for pRF parameters estimates for different methods. The correlation between the parameters estimated by the different methods (across all ROIs) is presented in the bottom row.

We evaluated the reliability of the pRF parameter estimates obtained with each of the approaches by computing the (Spearman) correlation (across voxels) of the pRF parameters estimated in two (independent) halves of the dataset (Figure 9). For the eccentricity and the pRF size we used the Spearman correlation to account for non-normal distributions while for the angle we used the circular correlation coefficient (Mardia, 1975). We performed this analysis in four regions of interest within the visual system: posterior, dorsal, lateral and ventral (see Benson et al., 2018 for more details on the definition of the regions) and in 181 subjects. We chose to perform the analysis within predefined regions of interest in order to be consistent with the analyses reported in Benson et al. (2018). In Figure 9 we report the results across the four ROIs combined. Note that, in order to compare our results to a previous analysis reported in Benson et al. (2018) we report here also the results obtained by the HCP7pRF approach using an exponent of 0.05 (HCP7pRF05). All methods except for linearized encoding showed similar reliability for the estimates of the pRF eccentricity and angle (i.e., the position in the visual field). In agreement with our simulations, the estimate of the pRF size obtained using model averaging was most reliable ($\rho_{\mathrm{pRF}\,\mathrm{size}}^{\text{ModelAve}}=0.81$ vs $\rho_{\mathrm{pRF}\,\mathrm{size}}^{\text{HCP}\,7\,\text{pRF}}=0.67$, mean values across the four ROIs, see Supplementary Table S5 for the results of the statistical comparisons between methods). Note that the reliability of the estimate of the pRF size obtained with the compressive non-linearity fixed to 0.05 was significantly lower than the one obtained using a fixed value of 1 (Supplementary Table S5). Linearized encoding resulted in the overall lowest reliability for the estimated pRF parameters (ρ_angle_ = 0.56,ρ_ecc_ = 0.83,ρ_*pRFsize*_ = 0.39, mean values across the four ROIs) and accuracy of the predictions (Supplementary Table S5). In terms of mean prediction accuracy all other methods performed similarly (prediction accuracy [correlation]: HCP7pRF05 = 0.37, HCP7pRF = 0.36, GS = 0.36, COpRF = 0.36, ModelAve = 0.36, LinEnc = 0.24). Note that despite the small effect size the small increase in prediction accuracy attainable when using a non-linear model (HCP7pRF05) was statistically significant (Supplementary Table S5).

By combining voxels across all regions of interest and averaging across all 181 subjects in the dataset, we computed the similarity (Spearman correlation) between the parameters estimated between the different methods (Figure 9 bottom row). All methods except linearized encoding resulted in similar estimates of the angle (with correlations above 0.85). Estimates of eccentricity were similar across all methods (with the minimum correlation of 0.79 between HCP7pRF05 and linearized encoding). As expected, the pRF size showed largest variability in its estimate across methods. Note that large correlation between the pRF parameters obtained with HCP7pRF and grid-search (above 0.9 for ecc, angle and the pRF size), which is an argument in favor of using grid-search over HCP7pRF considering their large difference in computational efficiency. The estimate of the pRF size obtained by fixing the compressive non-linearity to 0.05 was the least similar to all other approaches (that instead used a fixed value of 1).

### Auditory pRFs

**FIGURE 10:**
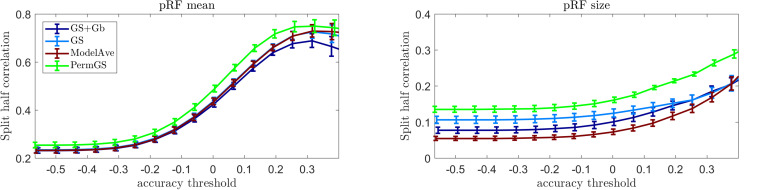
Reliability analysis for the auditory data. The mean reliability across 10 subjects is presented as a function of the mean prediction accuracy for four pRF estimation methods for the pRF mean (left) and size (central). In the right panel display the mean prediction accuracy (across 20000 voxels), for every subject.

Figure 10 shows the mean reliability (Spearman correlation between estimates obtained in two separate halves of the data) in the auditory data (10 subjects) as a function of the prediction accuracy. The pRF estimation using COpRF and LinEnc were not included in this analysis since these toolboxes were designed specifically for visual stimuli. We report reliability as a function of prediction accuracy as we hypothesize that evaluating the estimates of the model parameters should be limited to those voxels in which the model is a good descriptor of the response. As we are limiting the investigation to a simple model of sound frequency, high prediction accuracy is expected in early auditory cortical regions (Norman-Haignere and McDermott, 2018). It is relevant to note that previous studies (Santoro et al., 2014) have shown that feature spaces that are more complex than the use of only sound frequencies (e.g., the combination of frequency, spectral modulations and temporal modulations) may better describe the response in secondary cortical areas.

Our results indicate that the permutation based grid-search outperformed all other methods in terms of reliability of the estimated pRF mean and size. The statistical comparison of the reliability of the pRF mean and size across voxels whose prediction accuracy was larger than 0.2 (measured as the correlation between predicted and actual responses to the test sounds) is reported in Supplementary Table S6 (similar results were obtained when testing for differences between methods considering voxels thresholded at higher value of prediction accuracy). As when analyzing the visual data, also for the auditory data the two-steps optimization procedure (GS + Gb) was more computationally demanding (requiring over 24 h compared to the few minutes of GS alone). Note that the permutation approach will be more computationally demanding than the grid search alone. In particular considering a set of 42286 voxels, the permutation based approach was 21 times more expensive than grid search.

## Discussion and Conclusion

The use of computational approaches such as population receptive field modeling has become an established method for analyzing fMRI data (Gravel et al., 2014; Zuiderbaan et al., 2017; Dumoulin and Knapen, 2018). Despite the widespread application of this methodology, the analysis of *in vivo* data in a large sample (the Human Connectome) has highlighted low consistency in the estimate of the pRF size in comparison with the pRF mean (Benson et al., 2018). In this article we examined the influence of the optimization procedure on the variability of the pRF parameters and proposed modifications to the algorithms in order to improve the reliability of the pRF parameters in noisy scenarios. Following previous research, we considered as cost function the correlation and sum of squared errors. Both of these are strongly affected by outliers. The sensitivity to outliers of pRF estimation methods has not been considered in the literature, and in future studies it would be of interest to investigate pre-processing strategies or alternative cost functions that could limit such sensitivity.

The Gaussian model for the pRF and the cost function (correlation or minimum squared error) used to derive it define the optimization landscape. This optimization landscape, common to all estimation procedures, is at the basis of the large variability in the estimates of the pRF size that has been previously reported (Benson et al., 2018). In particular, estimates of the pRF size have higher variance because of the weaker sensitivity of the cost function to variations in the pRF size than to variations in the pRF mean. Here, we illustrate this issue using simulations (Figures 2, 3) as well as theoretically (Supplementary Material). We performed simulations at different noise levels and quantified SNR (of simulated and real data) using the split-half noise ceiling (an estimate of the maximum prediction accuracy that a pRF model can attain given the noise in the data) which is computationally efficient and is not based on assumptions about the noise structure (Lage-Castellanos et al., 2019). Computing the SHnc only requires that the data can be split in two identical partitions for computing the correlations between the observed responses.

When considering established pRF estimation procedures, our simulations showed that some approaches produce biased estimates of the pRF parameters across the different levels of noise and independently of the pRFs size (see the statistical comparisons reported in Table 1 as well as Supplementary Table S2). In particular, estimating pRFs with linearized encoding resulted in larger biases compared to all other approaches. With respect to the variability of the pRF size estimates previously reported (Benson et al., 2018), our simulations indicate that the difference in variability of the estimates between methods was more noticeable when considering narrow pRFs. For narrow pRFs, linearized approach produced more reliable estimates compared to grid search, HCP7pRF and COpRF at medium and low noise levels (Table 2). Taken together the analyses of the bias and variability in simulated data indicate that estimating pRFs with linearized encoding trades off bias in the pRF parameters for stability of the estimated pRF size. The weaker sensitivity of the cost function to changes in pRF size noticeable in simulated data was apparent also when estimating pRFs from real fMRI data obtained in response to visual stimulation (Figure 4). This weaker sensitivity resulted in different estimates of the pRF size when using independent splits of the fMRI data (Figure 5). Both the bias in the estimation of pRF parameters introduced by some of the approaches as well as the variability in the estimates of the pRF size may affect studies that aim at comparing the pRF size across brain regions or between experimental conditions. A bias in the estimated pRF parameters compromises accuracy and reproducibility especially in low SNR datasets (e.g., using high spatial resolution), while the increased variance of the estimates of the pRF size compromises statistical power. These issues highlight the relevance of controlling for the SNR in the data. Previous approaches have used a combination of heuristics to this end, ranging from smoothing the fMRI data to increase SNR (Dumoulin and Wandell, 2008) as well as considering only voxels (or regions) in which the pRF model explains a sufficient amount of variance in the signal (Dumoulin and Wandell, 2008; Thomas et al., 2015). In addition, constraints have been introduced allowing only pRFs broader than a certain value (Thomas et al., 2015; Zeidman et al., 2018). Interestingly, our results show that initiating the search for the pRF from regions of the parameter landscape with broad pRF may help obtain unbiased estimates (see e.g., Figure 3 where we show that the region of convergence for the optimization procedure is larger when the initial pRF size is set to larger values). When algorithms other than a simple grid-search are used, initial conditions for the optimization of pRF parameter are typically obtained from an initial grid-search. While in our simulations this resulted to be an effective strategy even in the case of low SNR, the development of an optimization strategy that moves specifically from broad to narrow pRFs could represent an interesting alternative for low SNR data (e.g., high resolution functional data) that to our knowledge has not been explored yet. It is important to note that here we used default hyperparameters values when using previously introduced estimation approaches (e.g., the regularization parameters in the ridge regression of the linearized encoding approach was fixed for all voxels to a value of equal to one). Fine-tuning hyperparameters may result in improved reliability of the estimated pRF parameters, but it is a data set specific problem that could be addressed in follow up studies. One interesting observation that derives from the results of our simulations is that performing a second optimization step (e.g., using Levenberg-Marquardt as in the implementation of the pRF method Benson et al., 2018) did not improve the reliability of the estimated parameters compared to grid-search (see Table 2 and Supplementary Table S3). This is particularly relevant considering the higher computational efficiency of grid-search compared to the rest of the algorithms (see Supplementary Table S1). We considered computational time for the estimation of the pRF of voxels with a medium level of noise (SHnc = 0.35). We expect this time to increase for all approaches except grid search when the noise in the data increases. It is plausible to assume that the absence of an improvement in the reliability of the estimates obtained with a second optimization step is caused by the effect of noise on the optimization routine resulting in non-replicable estimates. It is important to note that the computational complexity of grid-search increases rapidly with the number of pRF parameters to be estimated. In this article the number of parameters were limited to the pRF mean and size (e.g., the inclusion of the non-linearity term or the increase in dimensionality of the pRF mean).

With the aim of reducing the variability of the estimated pRF size in noisy scenarios here we introduced two specific modifications of the grid-search algorithm. It is important to note that, while these approaches improved the reliability of the pRF size estimates in simulated and real data, they do not constitute a principled solution to the low sensitivity of the cost function to the pRF size (as they are still based on the use of the same cost function). The first approach averages pRF models (obtained from grid-search) that result in similar goodness of fit to the data. This procedure can be interpreted as a Bayesian Model Average under a uniform prior. Here, we averaged models whose goodness of fit was within an interval of 1% of the minimum. The threshold of goodness of fit defining this interval has direct influence on the bias-variance tradeoff of the Model Average approach. Averaging more models will decrease the variance of the estimates but at the cost of increased bias. In addition, this hyperparameter should be carefully set depending on data quality. Noisier fMRI data will require averaging more models (i.e., considering a larger interval of goodness of fit) to achieve similar reductions of the variance of the estimates. How to calibrate this threshold conditioned to the noise in the data should be considered in the future. For narrow PRFs, model averaging resulted in improved reliability of the estimate of the pRF size compared to the previously introduced approaches we tested (Table 2). This improved reliability came at the cost of a small but significant bias in the estimate of the pRF parameters (Figure 8, Table 1 and Supplementary Table S2). A more systematic analysis of the reliability of the PRF size estimate in dependence with the pRF size indicated that using model averaging is advantageous when the pRF size generating the data is smaller than σ = 0.91. This indicates that using model averaging may be preferable when considering early visual cortical regions (see Figure 1 for a distribution of the pRF size in visual cortical areas in the HCP dataset). The improved reliability we observed in simulations was confirmed by the analysis of the HCP dataset, where model averaging resulted in a significant reduction of the variability of the pRF size estimates (Supplementary Table S5).

The second modification to the standard grid-search procedure that we introduce here is a permutation based procedure. This approach was motivated by the observation that, under the null hypothesis, the probability of obtaining a given fit is not independent of the pRF size. In particular, considering the two limit cases of infinite pRF size (i.e., a pRF with no preferred feature) and a pRF selecting a single feature, the first produces the same prediction regardless of the pRF mean, while pRFs selecting a single feature produce different (uncorrelated) predictions. As a result, the probability of selecting a narrow pRF model under the null hypothesis is higher than the probability of selecting a broader pRF model. The empirical null distribution of values of the cost function (e.g., correlation) for all the pRF parameters in a grid can be obtained using permutations. The selection of the pRF parameters is then based on the probability of observing under the null hypothesis a value of the cost function more extreme than that obtained in the original case. Importantly, the permutation procedure limits the applicability of this approach as it requires the observed data to be exchangeable under the null hypothesis, thus it can only be applied to particular stimulation designs (i.e., not in the case of the classical traveling wave retinotopic stimulation). While outside of the scope of our publication, it is worth noting that the experimental setup has been shown to have an effect on the estimation of pRF parameters even when controlling for the experimental time. The ordered presentation of tones (ascending and descending) resulted in higher goodness of fit to the observed fMRI data compared to a random presentation, and the random presentation of bars on the visual screen has been found to produce more reliable pRF estimates as evidenced by higher prediction accuracy (Senden et al., 2014). In addition, previous reports have in some cases discouraged the estimation of pRF size (bandwidth in the case of auditory pRFs) from ordered stimulus designs as it may be affected by the overemphasis of the response near the beginning or the end of the sweep (Thomas et al., 2015). These results justify the comparison of the permutation based approach to previously introduced estimation procedures on a design that like a random stimulus order design allows for the estimation of individual stimulus responses. In particular, we applied the permutation based approach to an auditory data in which natural sounds were presented and from which we estimated, per voxel, a vector representing the response to all the individual (natural) stimuli (Sitek et al., 2019). In this dataset, the permutation based approach resulted in improved reliability of the estimated pRF parameters (mean and size) compared to both model averaging and a two-step optimization procedure that has been previously introduced (Thomas et al., 2015). The improved reliability for the permutation approach came at the cost of computational time.

It is important to note that in all our investigations we considered a fixed non-linearity (fixed to 1 – a linear model) for the pRF model. We did not systematically consider the effect that either optimizing the non-linearity, or considering a term other than 1, would have on the estimates of the pRF mean and size. The simultaneous optimization of the pRF mean, size and non-linearity increases the number of local minima (Zeidman et al., 2018) and is expected to result in increased variability of the estimates. Here, when analyzing real visual data, we compared models estimated with an non-linearity fixed to one to the procedure previously reported that used a non-linearity fixed to 0.05 (Benson et al., 2018). Our results indicate that a non-linearity of 0.05 results in more variable estimates of the pRF parameters (angle, eccentricity and in particular size) despite a small advantage in prediction accuracy (Supplementary Table S5).

While our results on the validity of the estimates are in line with the results reported previously (Lerma-Usabiaga et al., 2020), here we focused on understanding how the interaction between the pRF model (e.g., Gaussian) and cost function underlie the previously reported variability of the pRF size. In this respect, while interesting, we did not consider the effect that the hemodynamic response function has on the estimates (which we considered fixed and equal across methods). The issues we highlight here of the sensitivity of the cost function to variations in the pRF size are thus general in nature and affect all estimation procedures similarly. This variability requires careful considerations when comparing the estimated pRF size across regions or experimental conditions. To limit this effect, previous approaches introduced heuristics based on imposing a threshold on the signal to noise ratio of the data or constraining the search of plausible pRF sizes. Here we introduce two modification of the grid-search optimization method based on model averaging and selecting the best model based on permutation testing. Both approaches showed improved reliability of the estimates of the pRF size in both simulated and real data.

## Data Availability Statement

Publicly available datasets were analyzed in this study. This data can be found here: https://db.humanconnectome.org/app/action/DownloadPackagesAction.

## Ethics Statement

The studies involving human participants were reviewed and approved by Ethics Review Committee Psychology and Neuroscience (ERCPN), Maastricht University. The patients/participants provided their written informed consent to participate in this study.

## Author Contributions

AL-C, GV, and FD performed the conceptualization. AL-C, GV, MS, and FD did the methodology and wrote, reviewed, and edited the manuscript. AL-C did the software, formal analysis, and wrote the original draft. All authors contributed to the article and approved the submitted version.

## Conflict of Interest

The authors declare that the research was conducted in the absence of any commercial or financial relationships that could be construed as a potential conflict of interest.

## Footnotes

1 https://db.humanconnectome.org/app/action/DownloadPackagesAction

2 https://osf.io/bw9ec/

3 https://cran.r-project.org/web/packages/circular/circular.pdf
