# Supplementary material for: Investigating the Reliability of Population Receptive Field Size Estimates Using fMRI
Source: Front Neurosci. 2020 Jul 30;14:825. doi: 10.3389/fnins.2020.00825 (PMC7408704; doi:10.3389/fnins.2020.00825)
Supplement: Supplementary file 1 [file Data_Sheet_1.pdf]

Supplementary Material

1 Supplementary text

1.1 Equivalence between the optimization problems defined by the cost functions $\boldsymbol{C_{SSE}}$ and $\boldsymbol{C_{1-corr}}$.

Starting from the definitions of C_{SSE} and C_{1-corr}:

$$C_{SSE} = \left(\boldsymbol{y} - \alpha\boldsymbol{X}\boldsymbol{w}(\boldsymbol{\mu}, \sigma)\right)^T\left(\boldsymbol{y} - \alpha\boldsymbol{X}\boldsymbol{w}(\boldsymbol{\mu}, \sigma)\right)$$

$$C_{1-corr} = 1 - \frac{\boldsymbol{y}^T\boldsymbol{X}\boldsymbol{w}(\boldsymbol{\mu}, \sigma)}{\sqrt{\boldsymbol{w}^T\boldsymbol{X}^T\boldsymbol{X}\boldsymbol{w}}}$$

C_{SSE} can be expanded as:

$$C_{SSE} = \left(\boldsymbol{y} - \alpha\boldsymbol{X}\boldsymbol{w}(\boldsymbol{\mu}, \sigma)\right)^T\left(\boldsymbol{y} - \alpha\boldsymbol{X}\boldsymbol{w}(\boldsymbol{\mu}, \sigma)\right)$$

$$C_{SSE} = \boldsymbol{y}^T\boldsymbol{y} - \boldsymbol{2}\alpha\boldsymbol{y}^{\boldsymbol{T}}\boldsymbol{X}\boldsymbol{w} + \alpha^2\boldsymbol{w}^{\boldsymbol{T}}\boldsymbol{X}^{\boldsymbol{T}}\boldsymbol{X}\boldsymbol{w}$$

Defining the scaling parameter as $\alpha^2 = \frac{1}{\boldsymbol{w}^{\boldsymbol{T}}\boldsymbol{X}^{\boldsymbol{T}}\boldsymbol{X}\boldsymbol{w}}$

$$C_{SSE} = \boldsymbol{y}^T\boldsymbol{y} - \boldsymbol{2}\frac{\boldsymbol{y}^{\boldsymbol{T}}\boldsymbol{X}\boldsymbol{w}}{\sqrt{\boldsymbol{w}^{\boldsymbol{T}}\boldsymbol{X}^{\boldsymbol{T}}\boldsymbol{X}\boldsymbol{w}}} + 1$$

$$C_{SSE} = \boldsymbol{y}^T\boldsymbol{y} - 2\frac{\boldsymbol{y}^{\boldsymbol{T}}\boldsymbol{X}\boldsymbol{w}}{\sqrt{\boldsymbol{w}^{\boldsymbol{T}}\boldsymbol{X}^{\boldsymbol{T}}\boldsymbol{X}\boldsymbol{w}}} + 2 - 1$$

$$C_{SSE} = \boldsymbol{y}^T\boldsymbol{y} + 2C_{1-corr} - 1$$

This equivalence is valid under the assumption that $\boldsymbol{y}$ and the columns of $\boldsymbol{X}$ have zero mean.

1.2 Derivatives of the cost function respect to $\boldsymbol{\mu}$ and $\boldsymbol{\sigma}$.

The simplest form of computing these derivatives is using the chain rule:

$$\frac{\partial C}{\partial \mu_j} = \sum_i^f \frac{\partial C}{\partial w_i}\frac{\partial w_i}{\partial \mu_j} \qquad \frac{\partial C}{\partial \sigma} = \sum_i^f \frac{\partial C}{\partial w_i}\frac{\partial w_i}{\partial \sigma}$$

Where f defines the total number of features in the pRF model (pixels for the visual case or frequencies for auditory case), i refers to the components of the gaussian vector and j refers to dimensions in the Gaussian (for visual data: x, y). The derivatives of the Gaussian function respect to the pRF parameters are:

$$\frac{\partial w_i}{\partial \mu_j} = \alpha w_i \frac{\left(r_{ij} - \mu_j\right)}{\sigma^2} \qquad \frac{\partial \boldsymbol{w}}{\partial \sigma} = \alpha w_i \frac{1}{\sigma^3}(\boldsymbol{r_i} - \boldsymbol{\mu})^T(\boldsymbol{r_i} - \boldsymbol{\mu})$$

Derivatives of the cost function respect to the Gaussian vector are:

$$\frac{\partial C_{SSE}}{\partial \boldsymbol{w}} = -2\boldsymbol{X}^T(\boldsymbol{y} - \boldsymbol{Xw})$$

$$\frac{\partial C_{1-corr}}{\partial \boldsymbol{w}} = -\frac{\boldsymbol{X^T y}}{\sqrt{\boldsymbol{w}^T\boldsymbol{X}^T\boldsymbol{Xw}}} + \frac{\boldsymbol{X}^T\boldsymbol{Xw}}{(\boldsymbol{w}^T\boldsymbol{X}^T\boldsymbol{Xw})^{3/2}}\boldsymbol{y}^T\boldsymbol{Xw}$$

1.3 Limits of the derivatives of the cost function when $\boldsymbol{\sigma} \to \mathbf{0}$

In this section we analyze the limits: $\lim_{\sigma\to 0}\frac{\partial C_{SSE}}{\partial \mu_j}$ and $\lim_{\sigma\to 0}\frac{\partial C_{SSE}}{\partial \sigma}$. The chain rule is used to expand the derivatives in terms that are then considered in the limit case. We present the analysis for C_{SSE} however a similar approach can be used for C_{corr}. The derivatives of the cost function with respect to the pRF parameters $\boldsymbol{\mu}$ and σ are:

$$\frac{\partial C}{\partial \mu_j} = \sum_{i}^{f} \frac{\partial C}{\partial w_i}\frac{\partial w_i}{\partial \mu_j} \qquad\qquad \frac{\partial C}{\partial \sigma} = \sum_{i=1}^{f} \frac{\partial C}{\partial w_i}\frac{\partial w_i}{\partial \sigma}$$

The two terms inside the sum are the derivatives of the cost function respect to $\boldsymbol{w}$: $\frac{\partial C_{SSE}}{\partial \boldsymbol{w}} = -2\boldsymbol{X}^T(\boldsymbol{y} - \boldsymbol{X}\boldsymbol{w})$ and the derivatives of the Gaussian weights $\boldsymbol{w}$ with respect to the pRF mean and pRF size: $\frac{\partial w_i}{\partial \mu_j}$, $\frac{\partial w_i}{\partial \sigma}$ (see previous section).

The first term in the derivatives is of the form $\frac{\partial C}{\partial w_i} = \sum_k^n X_{k,i} e_k$, where e_k are the errors of the pRF model for the sample k. The limits of these terms when $\sigma \to 0$ are influenced by the error of the model when the pRF gets narrower, but the important aspect is that these values are bounded. Instead, the limit when $\sigma \to 0$ of the terms $\frac{\partial w_i}{\partial \mu_j}$ and $\frac{\partial w_i}{\partial \sigma}$ is zero. Considering the derivatives as obtained in the previous section, the limit when $\sigma \to 0$ of the terms $\frac{\partial w_i}{\partial \mu_j}$ and $\frac{\partial w_i}{\partial \sigma}$ can be expressed as:

$$\lim_{\sigma \to 0_+} \frac{u}{\sigma^2} e^{-\frac{u^2}{\sigma^2}} \qquad\qquad \lim_{\sigma \to 0_+} \frac{u^2}{\sigma^3} e^{-\frac{u^2}{\sigma^2}}$$

Note that $\frac{u}{\sigma^2} e^{-\frac{u^2}{\sigma^2}}$ and $\frac{u^2}{\sigma^3} e^{-\frac{u^2}{\sigma^2}}$ represent a Gaussian multiplied by a polynomial function (respect to u). The Gaussian component approaches the delta function when $\sigma \to 0$, while the polynomials approach the mean of the gaussians ($u = 0$) when $\sigma \to 0$. As a result, the limits of both derivatives of the cost functions respect to the pRF parameters are zero.

2 Supplementary Figures and Tables

2.1 Supplementary Figures

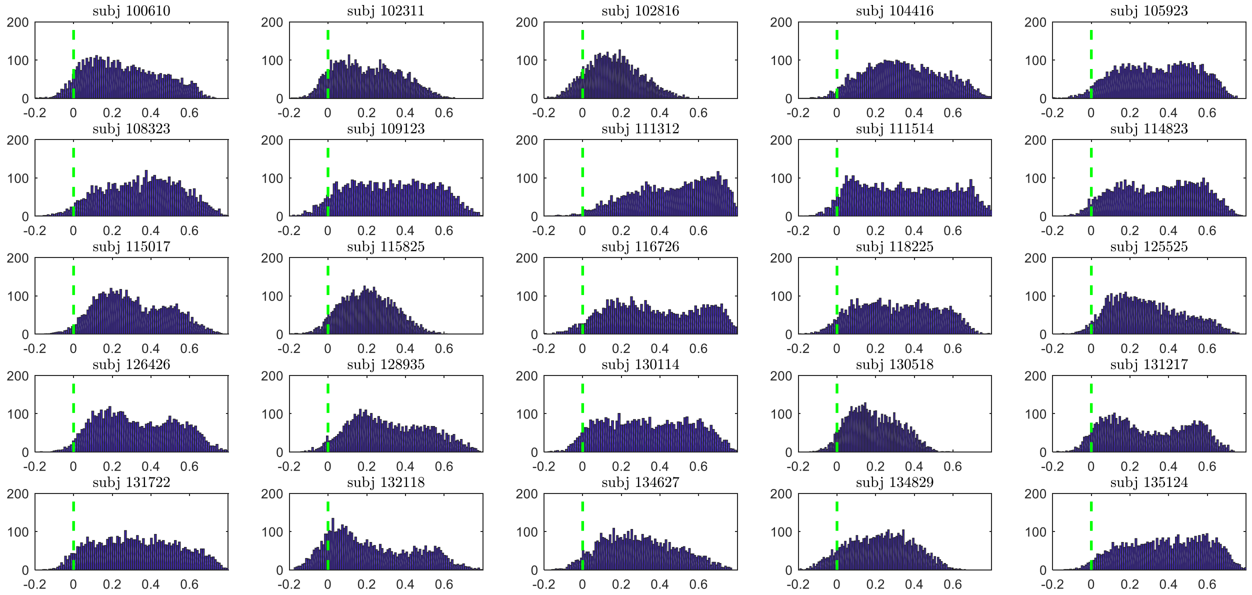

Supplementary Figure 1. Histograms of the voxelwise SHnc for 25 subjects in the HCP dataset. The SHnc was obtained by correlating the voxel's time series of fMRI runs 5 and 6 (both fMRI runs have the identical experimental design).

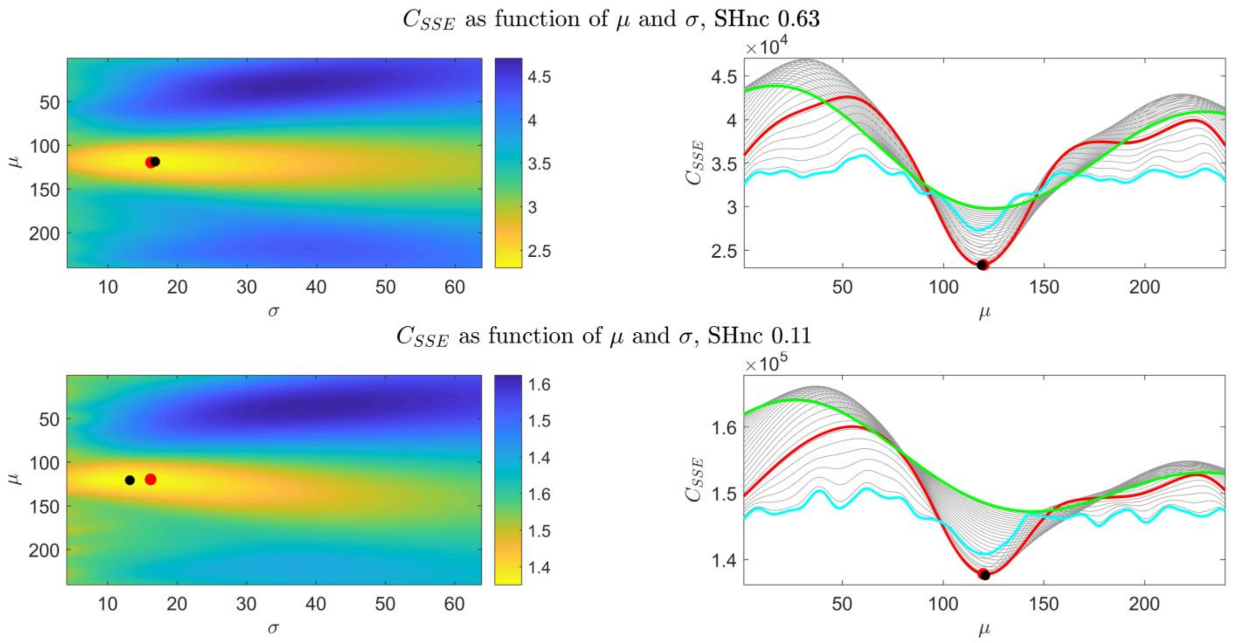

Supplementary Figure 2. Cost function landscape for C_{SSE} as a function of μ and σ. Upper and bottom left panels show C_{SSE} as a function of μ and σ for high and low SHnc respectively. The right panels show the corresponding profiles with respect to μ, while the different (gray) curves are obtained with different σ values. The values of C_{SSE} as a function of μ for the broadest and narrowest possible pRFs are displayed in green and cyan respectively. The values of C_{SSE} as a function of μ for the pRF that generated the data are depicted in red. Red dots and black dots denote the μ, σ used to generate the data and the global minimum respectively. In the high SHnc scenario the generative pRF (red) and the global minimum (black) are in the same location in the (μ, σ) space (the dots are superimposed).

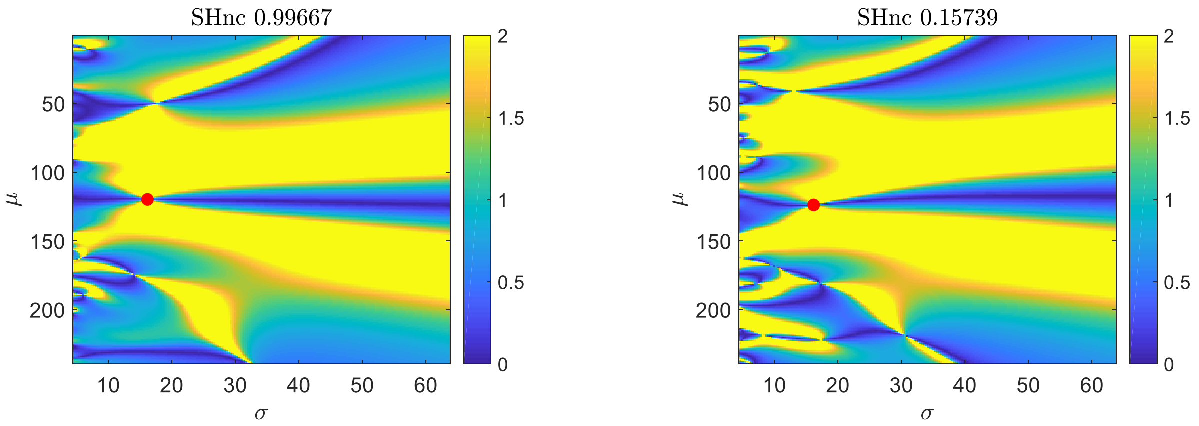

Supplementary Figure 3. Ratio between the derivatives $\frac{\partial C_{corr}}{\partial \mu}$ and $\frac{\partial C_{corr}}{\partial \sigma}$ for two realizations of the one dimensional Gaussian simulation with high (left) and low (right) SHnc values. The values of the pRF parameters that generated the data are depicted by the red dot. Note in the figure that the region around the true pRF parameters (area in yellow) is dominated by the derivative respect to the mean (e.g the ratio $\frac{\partial C}{\partial \mu}/\frac{\partial C}{\partial \sigma} > 1$). The horizontal blue line located at the true pRF mean ($\mu = 120$) denotes the curve where the derivative respect to μ is null and the quotient becomes infinite. In the regions where $\frac{\partial C}{\partial \mu}/\frac{\partial C}{\partial \sigma} < 1$ (blue color) the pRF size (σ) has larger influence on the cost function than the pRF mean μ. Otherwise, in the regions where $\frac{\partial C}{\partial \mu}/\frac{\partial C}{\partial \sigma} > 1$ (yellow) the pRF mean has larger influence on the cost function than the pRF size.

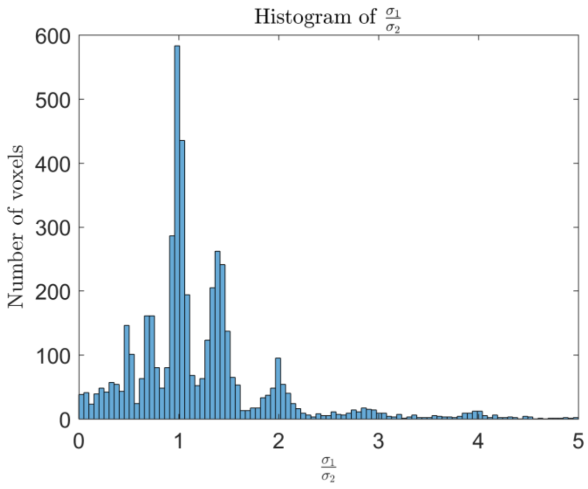

Supplementary Figure 4. Ratio between the estimated pRF sizes in two independent splits of the data from subject 100610 in the HCP dataset. The histogram comprises all voxels in the visual cortex.

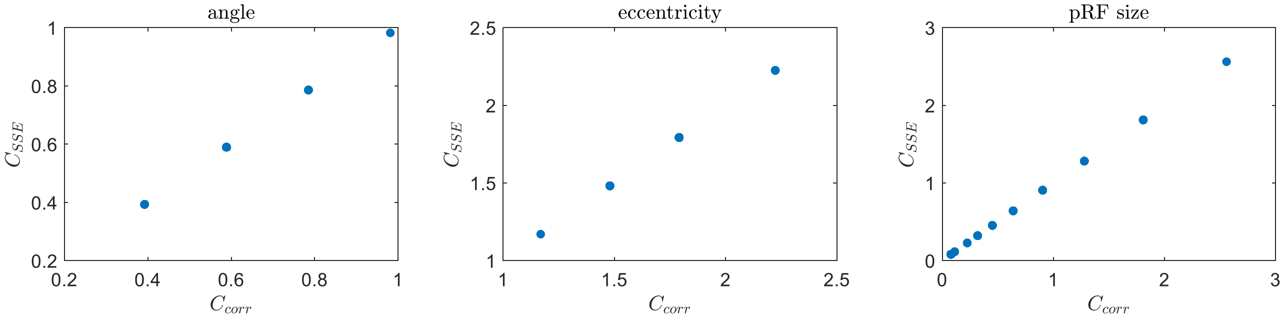

Supplementary Figure 5. Comparison between the pRF parameters estimated using $\mathrm{C_{SSE}}$ and $\mathrm{C_{corr}}$. PRF parameters obtained using C_{SSE} vs pRF parameters obtained using $\mathrm{C_{corr}}$. The pRF parameters were obtained using grid search under two different cost functions. The pRF size used for simulating the data was incrementally increased and for each pRF size the simulation was repeated 1000 times (see Figure 8 description). This comparison confirms that grid search under $\mathrm{C_{SSE}}$ and $\mathrm{C_{corr}}$ produced identical values of the pRF parameters.

Supplementary Table 1. Runtime analysis on simulated visual responses. CPU time is presented relative to grid-search (ratioGS – this choice is motivated by the fact that GS was the least computationally demanding procedure).

Number of voxels	HCP7pRF ratioGS	COpRF ratioGS	LinEnc ratioGS	ModelAve ratioGS
1000	234.9	4.9	1.4	1.7
2000	446.6	11.0	2.4	3.1
3000	657.0	13.4	3.4	2.9
4000	830.0	16.3	4.3	3.5
5000	1016.9	20.9	5.1	4.0

Supplementary Table 2. Statistical analysis of the bias of the estimates of the angle, eccentricity and pRF size obtained when simulating a broad visual pRF (angle = 0.59 radians, eccentricity = 1.79^{o}, pRF size $\sigma = 1.81^{o}$) at different noise levels. For every noise level, columns represent the different parameters while rows represent the different estimation methods. For significant effects ($p < 0.05$) we report the effect size (in units of the parameter), while n.s. indicates a non-significant effect. When significant bias was observed, the corresponding cell displays the mean of the estimated parameter minus the its true value.

	**Low Noise**			**Medium Noise**			**High Noise**		
	Angle	*Ecc.*	*Size*	*Angle*	*Ecc.*	*Size*	*Angle*	*Ecc.*	*Size*
HCP7pRF	0.003	n.s.	n.s.	n.s.	n.s.	n.s.	n.s.	n.s.	0.014
GS	n.s.	n.s.	n.s.	n.s.	n.s.	n.s.	n.s.	n.s.	n.s.
COpRF	n.s.	-0.11	-0.05	n.s.	-0.11	0.04	n.s.	-0.06	n.s.
LinEnc	n.s.	-0.57	-0.66	n.s.	-0.56	-0.74	n.s.	-0.58	-0.81
ModelAve	n.s.	-0.05	-0.01	n.s.	n.s.	0.01	n.s.	n.s.	0.01.

Supplementary Table 3 Pairwise comparison of the variability of the estimates of the pRF size between estimation methods (broad pRF: angle = 0.59 radians, eccentricity = 1.79°, pRF size σ = 1.81°). Every cell displays (for significant effects) the variance of the pRF size estimated by the method in the row divided by the variance of the method in the column ($\frac{Var(\sigma)_{row}}{Var(\sigma)_{col}}$), while n.s. indicates a non- significant effect.

	**Low Noise**				**Medium Noise**				**High Noise**			
	GS	***COpRF***	***LinEnc***	***ModelAve***	***GS***	***COpRF***	***LinEnc***	***ModelAve***	***GS***	***COpRF***	***LinEnc***	***ModelAve***
HCP7pRF	n.s.	0.14	0.007	0.46	n.s.	0.59	0.06	n.s.	n.s.	1.13	0.61	0.79
GS		n.s.	n.s.	n.s.		0.55	0.06	1.3		1.14	n.s.	0.81
COpRF			0.05	3.27			0.11	12.36			0.62	0.76
LinEnc				63.43				n.s.				n.s.

Supplementary Table 4. Pairwise comparison of the variability of the estimates of the pRF size between estimation methods for a medium noise scenario (SHnc = 0.35) in simulated visual data. The results are reported for pRFs of different sizes (σ) and is in reference to the results reported in Figure 8. Every cell displays (for significant effects) the variance of the pRF size estimated by the method in the row divided by the variance of the estimates obtained with model averaging ($\frac{\boldsymbol{Var(\sigma)_{row}}}{\boldsymbol{Var(\sigma)_{MAve}}}$), while n.s. indicates a non significant effect.

	**σ =0.08**	**σ =0.11**	**σ =0.16**	**σ =0.23**	**σ =0.32**	**σ =0.64**	**σ =0.91**	**σ =1.28**	**σ =1.81**
**HCP7pRF**	3.88	10.51	7.49	11.89	5.94	2.59	1.86	0.64	0.57
**GS**	3.95	10.38	7.28	11.75	5.98	2.57	1.87	0.66	0.62
**COpRF**	3.45	9.32	6.76	14.27	6.14	2.76	1.74	0.55	0.44

Supplementary Table 5. Pairwise comparison of the reliability (measured with split half correlation) of the estimates of the pRF parameters in the HCP dataset. Every cell represents the difference between the method reported in the row and the method reported in the column (i.e. a positive number indicates that the method in the row is more reliable than the method in the column). For significant effects ($p < 0.05$) we report the difference in reliability (averaged across regions of interests and subjects).

	**Angle**					**Eccentricity**				
	HCP7pRF	***GS***	***COpRF***	***LinEnc***	***MAve***	***HCP7pRF***	***GS***	***COpRF***	***LinEnc***	***MAve***
HCP7pRF05	-0.001	n.s.	-0.008	0.27	-0.001	0.007	-0.01	n.s.	0.06	n.s.
HCP7pRF		0.0008	-0.006	0.27	n.s.		-0.004	n.s.	0.06	0.004
GS			-0.007	0.27	-0.0004			0.008	0.07	0.008
COpRF				0.27	0.006				0.05	n.s.
LinEnc					-0.27					-0.06

	**pRF size**					**Accuracy**				
	HCP7pRF	***GS***	***COpRF***	***LinEnc***	***MAve***	***HCP7pRF***	***GS***	***COpRF***	***LinEnc***	***MAve***
HCP7pRF05	-0.20	-0.22	-0.20	0.15	-0.33	0.005	0.006	0.004	0.12	0.005
HCP7pRF		-0.01	n.s.	0.36	-0.11		0.0003	-0.001	0.11	-0.0002
GS			0.01	0.38	-0.09			-0.001	0.11	-0.0006
COpRF				0.36	-0.12				0.11	0.001
LinEnc					-0.49					-0.11

Supplementary Table 6. Pairwise comparison of the reliability of the estimates of the pRF parameters in the auditory dataset. The analysis refers to reliability measured across all voxels whose prediction accuracy is higher than 0.2 (see Figure 10). Every cell represents the difference between the method reported in the row and the method reported in the column (i.e. a positive number indicates that the method in the row is more reliable than the method in the column). For significant effects ($p < 0.05$) we report the difference in reliability (averaged across regions of interests and subjects).

	**pRF mean**			**pRF size**		
	GS	***ModelAve.***	***PermGS***	***GS***	***ModelAve.***	***PermGS***
GS+Gb	-0.02	-0.02	-0.08	*n.s*	0.02	-0.07
GS		*n.s*	-0.05		0.04	-0.06
ModelAve			-0.06			-0.9
